# Morphological examination of the visual system and orbital region in the red panda (*Ailurus fulgens fulgens*)

**DOI:** 10.1186/s12917-024-04152-2

**Published:** 2024-07-02

**Authors:** Joanna E. Klećkowska-Nawrot, Karolina Goździewska-Harłajczuk, Karolina Barszcz, Krzysztof O. Stegmann

**Affiliations:** 1 Department of Biostructure and Animal Physiology, Faculty of Veterinary Medicine, Wroclaw University of Environmental and Life Sciences, Kozuchowska 1, Wroclaw, 51-631 Poland; 2 Department of Morphological Sciences, Institute of Veterinary Medicine, Warsaw University of Life Sciences, Nowoursynowska 159, Warsaw, 02-787 Poland; 3 GeoWild, Marsz. Jozefa Pilsudskiego 74 lok. 320, Wroclaw, 50-020 Poland

**Keywords:** Eyelids, Orbital gland, Eye tunics, Superficial gland of the third eyelid, Lacrimal gland

## Abstract

**Objectives:**

The red panda is currently the only surviving member of the Ailuridae family in the Caniformia suborder. In this study, we provide data on anatomical, morphometric, histological and histochemical examination of the orbital region, eyelids, orbital gland, and eye tunics in two adult males *Ailurus fulgens fulgens* from the Wroclaw Zoological Garden, Poland.

**Methods:**

The study involved morphometric analysis of the eyeball and selected accessory organs of the eye, along with analysis of the bony orbit, including its morphometry, macroscopic, and microscopic evaluation. Microscopic evaluation encompassed histological and histochemical staining, with the former involving hematoxylin & eosin (H&E), Movat pentachrome, picro-Mallory trichrome, Fontana-Masson, and the latter including PAS, AB pH 1.0, AB pH 2.5; AB pH 2.5/PAS, and HDI.

**Results:**

The upper (UE) and lower (LE) eyelids presented well-developed tarsal glands, sebaceous glands, and a characteristic simple alveolar gland (producing a mucous secretion). The palpebral part of the lacrimal gland was present. A single lymphoid follicle was observed only in the upper eyelids. The superficial gland of the third eyelid (SGTE) was a multilobar acinar complex that produces mucous secretion and is contained within the interlobular ducts of numerous aggregates of lymph nodes. The third eyelid (TE) was T-shaped and composed of hyaline tissue, containing CALT. The lacrimal gland (LG) also revealed a multilobar acinar complex that produced mucous secretion, with a single lymphoid follicle. The cornea consisted of 4 layers, as Bowman’s membrane was absent. The Vogt palisades composed of 7–10 layers of epithelial cells were demonstrated. The pupil was horizontally ovoid at rest (*post-mortem*). The sphincter pupil and the dilator pupil were well developed. Macroscopically, the *tapetum lucidum* appeared as a milky, non-opalescent crescent. Histologically, the choroidal *tapetum lucidum cellulosum* consisted of 5 to 9 layers of loosely packed oval cells. The retina showed a composition similar to that of terrestrial nocturnal carnivores.

**Conclusions:**

The results of our research indicate that the anatomical features of the eye and orbital region in the red panda share similarities with those described in the Musteloidea clade, as well as the Canidae and Ursidae families.

## Introduction

The red panda (*Ailurus fulgens*) is the only member of the unique Ailuridae family and is an endangered mammal endemic to the Himalayas [1–5]. Hu et al. [3] rejected the Nujiang River as the boundary of species distribution and proposed the Yalu Zangbu River as a geographic barrier between the Chinese red panda, found in southeastern Tibet and northern Myanmar, and the Himalayan red panda, found in Nepal and southern Tibet [3].

Based on morphological studies that indicate differences in skull anatomy, coat color, tail ring, and geographic distribution, two subspecies of red pandas have been distinguished: the Himalayan (*Ailurus fulgens fulgens* Cuvier, 1825) and the Chinese subspecies (*Ailurus fulgens styani* Thomas, 1902) [3, 6–8]. The Chinese panda is characterized by a greater skull length, a much larger zygomatic breadth, prominent coronoid processes in the mandible, stronger frontal convexity due to more extensive frontal sinuses, a redder face coat color with less white or more distinct tail rings [1, 3, 6, 8–10]. *Ailurus* sp. has small, dark-colored eye patches [11] with small, bear-like eyes [12]. Compared to other carnivores of similar size, such as Procyonidae, the skull of the red panda is larger [5, 13, 14].

Wild red pandas are primarily active at dawn, dusk, and night [5, 6, 15–17], whereas captive individuals tend to be nocturnal and crepuscular, sleeping during the day in trees and exhibiting a polyphasic activity pattern throughout the night [5, 6]. However, red pandas are more active during daylight, especially in summer that coincides with arboreal foraging, and rest in direct sunlight during winter to minimize heat loss [11]. Keller [18] and Roberts [19] report that activity patterns change throughout the year in response to temperature, feeding regimes, and the presence of young [18, 19].

Unfortunately, the literature lacks details about red pandas’ visual abilities. Bartlett [12] described their eyes as small and bear-like. Research on color vision capacity was conducted among Procyonidae, describing diurnal ring-tailed coatis (*Nasua nasua*) as dichromatic and nocturnal kinkajous (*Potos flavus*) and common raccoons (*Procyon lotor*) as monochromatic [20–30]. Similar research was carried out among Ursidae, including the giant panda (*Ailuropoda melanoleuca*), American black bear (*Ursus americanus*), brown bear (*Ursus arctos*), and the polar bear (*Ursus maritimus*), all of which are dichromatic, as other carnivores that are not strictly nocturnal [31–33].

The literature provides the correct skull structure and anatomical description of the orbital region, accessory eye organs, and eyeball at the macro and microscopic levels, but clinical cases related to eye pathology in the Ailuridae family are scarce [1, 3, 5, 34–43].

This study represents the first investigation into the anatomical, histological, and histochemical aspects of the red panda *Ailurus fulgens fulgens’* orbital region, eyelids, orbital glands, and eyeball, Additionally, our research aims to compare macro- and micromorphological findings of the selected ocular structures with those available in the literature for other species within the Musteloidea clade, but also with those of the Canidae and Ursidae families, to reveal similarities and differences among these animals. This work can also serve as a valuable source of information for veterinarians working in national parks and zoos, providing diagnostic tests and surgical procedures for eye conditions in this only living representative of the Ailuridae family.

## Materials and methods

### Animals

**Fig. 1:**
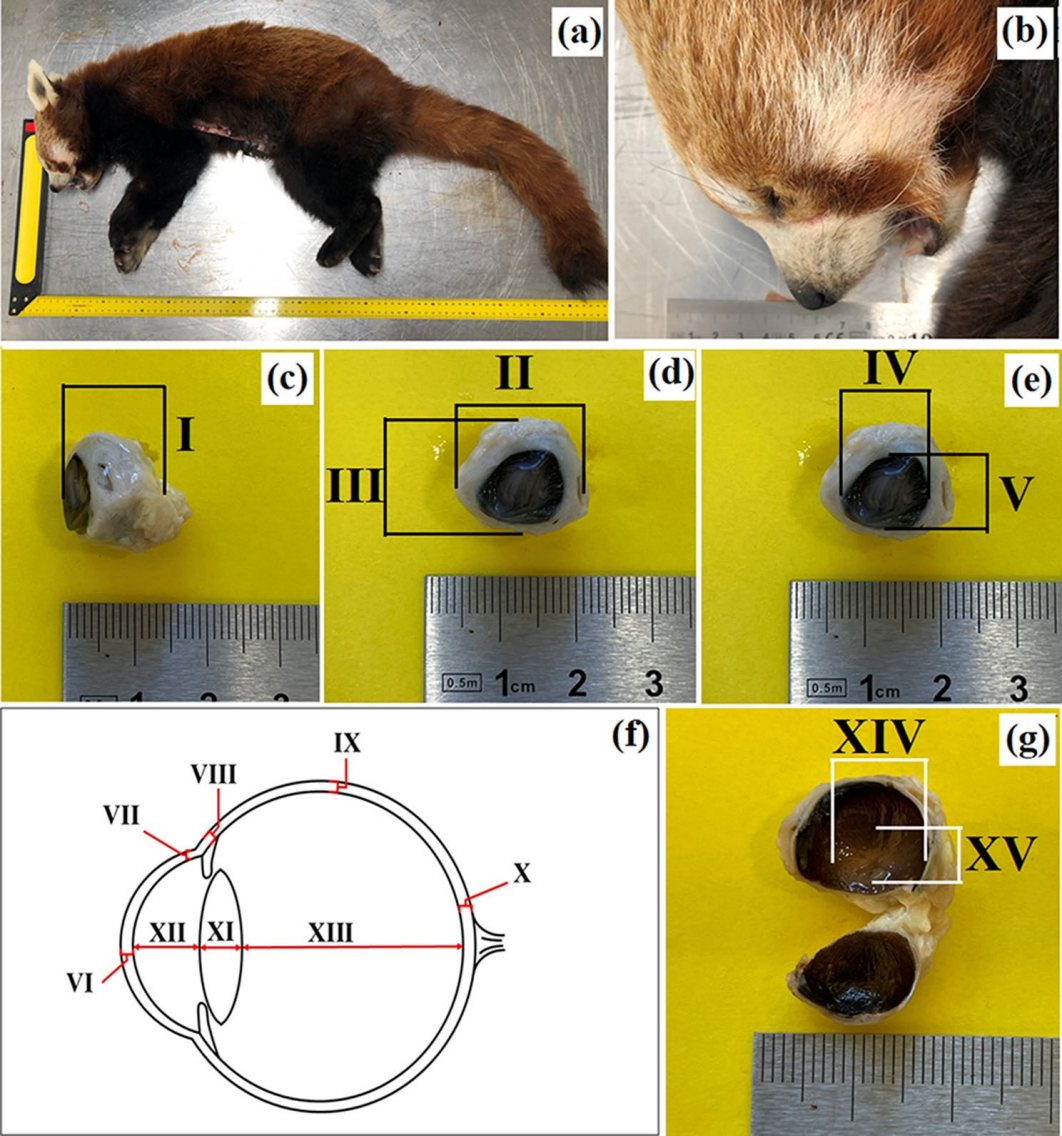
The photomacrograph of the *Ailurus fulgens fulgens* (**a**, **b**) and parameters of the eyeball (**c** – **g**): (**c**) I – axial eye diameter; (**d**) II – maximum transverse (equatorial) eye diameter, III – minimum transverse (equatorial) eye diameter; (**e**) IV – maximum corneal diameter, V – minimum corneal diameter; (**f**) VI – corneal axial thickness, VII – corneal peripheral thickness, VIII – scleral limbus thickness, IX – scleral equator thickness, X – scleral optic nerve thickness, XI – lens axial length, XII – aqueous chamber depth, XIII – vitreous chamber depth; (**g**) XIV – *tapetum lucidum* length, XV – *tapetum lucidum* width

The study was conducted on two male captive red pandas (*A. f. fulgens*) from Wroclaw Zoological Garden (Poland) (Fig. 1a and b). The first male, named Yunnan, passed away on May 29th, 2020, at the age of 11 years, 11 months, and 6 days, due to natural causes. The second male, named Lapi, passed away on December 17th, 2020, at the age of 1 year, 4 months, and 28 days, due to pneumonia.

### Ethical statement

For the acquisition of *post-mortem* materials, personal permits were obtained from the District Veterinary Officer in Wroclaw (Poland) (No. PIW Wroc. UT-45/5/16 - Dr. Joanna Klećkowska-Nawrot, No. PIW Wroc. UT-45/6 /16 - Dr. Karolina Goździewska-Harłajczuk). According to Polish and European law, studies on tissues obtained *post-mortem* do not require approval from the Ethics Committee (Directive 2010/63/EU of the European Parliament and of the Council of September 22nd, 2010, and the Journal of Laws of the Republic of Poland, the Act of January 15th, 2015, on the protection of animals used for scientific or educational purposes).

### Measurements and conservation of the eyeballs and accessory organs of the eye

First, macroscopic measurements of UE (*n* = 4) and LE (*n* = 4) were performed, followed by the collection of these tissues. Subsequently, eyeballs (*n* = 4), SGTE (*n* = 4), TE (*n* = 4), and LG (*n* = 4) were collected. All samples were obtained immediately after the animals died and placed in plastic containers filled with 4% buffered formaldehyde.

Eyeballs were measured following the method described by Hermanson et al. [44] (Fig. 1c and g): axial eye diameter: from the anterior cornea to the optic disc; maximum transverse (equatorial) eye diameter; minimum transverse (equatorial) eye diameter; maximum corneal diameter; minimum corneal diameter; corneal - axial – thickness; corneal - peripheral – thickness; scleral - limbus – thickness; scleral - equator – thickness; scleral - optic nerve – thickness; lens - axial length; aqueous chamber depth; vitreous chamber depth; *tapetum lucidum* length; *tapetum lucidum* width; optic nerve diameter.

Macroscopic measurements (length, width, and thickness) of the eyelids and orbital glands were taken using a digital caliper with a resolution of 0.01 mm and an accuracy of +/- 0.02 mm (> 100 mm) (Handy Worth, Poland), following the method described by Klećkowska-Nawrot et al. [45]. The values of six randomly selected measurements of the whole orbit, eyelids, orbital glands, and eyeballs were statistically analyzed for mean, standard deviation (SD), and coefficient of variation (CV).

### Anatomical description and measurements of the orbit

**Fig. 2:**
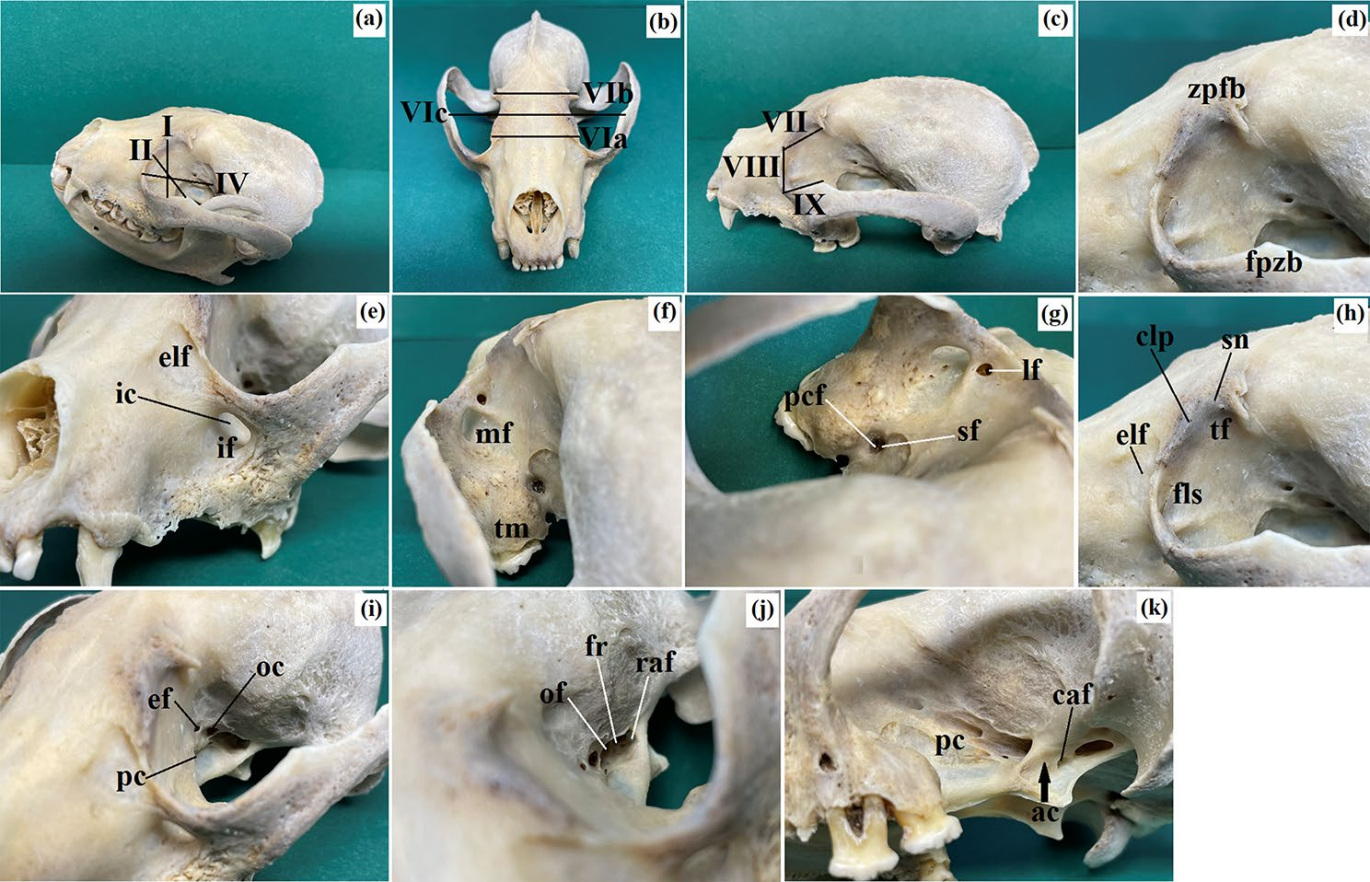
The photomacrograph of the *Ailurus fulgens fulgens* orbital region: (**a**) I – orbital vertical length, II – orbital horizontal width, IV – orbital depth; (**b**) VIa – at rostral level, VIb – at middle level, VIc – at caudal level; (**c**) VII – frontal length, VIII – lacrimal length, IX – malar length; (**d**) fpzb – frontal process of zygomatic bone, zpfb – zygomatic process of frontal bone; (**e**) elf – external lacrimal fossa, ic – infraorbital canal, if – infraorbital foramen; (**f**) mf – maxillary foramen, tm – tuberosity of maxilla; (**g**) lf – lacrimal foramen, pcf – palatine caudal foramen, sf – sphenopataline foramen; (**h**) clp – caudal lacrimal process, elf – external lacrimal fossa, fls – fossa for lacrimal sac, sn – supraorbital notch, tf – trochlear fovea; (**i**) ef – ethmoid foramen, oc – optic canal, pc – pterygoid crest; (**j**) fr – foramen rotundum, of – orbital fissure, raf – rostral alar foramen; (**k**) ac – alar canal, caf – caudal alar foramen, pc – pterygoid crest

The osteological description of the orbital region was conducted following the methodology outlined by Nickel et al. [46] and *NAV* [47]. The orbital morphometry was described according to the method described by Hermanson et al. [44] (Fig. 2a and c; Table 1). Macroscopic images were captured with a Nikon D300s camera equipped with a Tamron AF 17–50 mm F/2.8 [IF] ∅ 67 lens.

**Table 1:** Orbital measurement parameters

Parameters		Description
orbital vertical length (mm):		the perpendicular distance between the supraorbital and infraorbital margins of the orbit
orbital horizontal width (mm):		the distance between the rostral and caudal margins of the orbital rim
orbital index (%)		(orbital width (II)×100%)/(orbital length (I))
orbital depth (mm)		distance between the optic foramen and the center of the orbital rim
orbital area (mm^2^)		22/7 x (½ I x ½ II)
interorbital distance (mm)	at rostral level	distance between the junction of the frontolacrimal sutures on either side at the rostral margin of the orbit
	at middle level	distance between the supraorbital borders of the orbit on either side
	at caudal level	distance between the junctions of the zygomatic bone at the caudal margin of the orbit on either side
frontal length (mm)		distance from the tip of the zygomatic process of the frontal bone to the frontolacrimal sutures
lacrimal length (mm)		distance from frontolacrimal sutures to the junction between the lacrimal and zygomatic bones
malar length (mm)		distance from the junction between the lacrimal and zygomatic bones to the tip of the frontal process of the zygomatic bones

### Processing and histological evaluation of the eyeball and accessory organs of the eye

The eyelids, orbital glands, and eyeballs were placed in 4% buffered formaldehyde for 72 h and then rinsed in running water for 24 h. Subsequently, they were processed using a vacuum tissue processor – ETP (RVG3, Intelsint, Italy) and embedded in paraffin. The samples were sectioned into 4 μm slices using a Slide 2003 sliding microtome (Pfm AG, Germany). The following staining methods were applied: hematoxylin & eosin, Movat pentachrome (modified Russell Movat), picro-Mallory trichrome, and Fontana-Masson. The resulting slides were observed using a Zeiss Axio Scope A1 light microscope (Carl Zeiss, Jena, Germany) and rated using a scoring system based on a standard protocol as previously described [48, 49]. *NAV* [47] and *NHV* [50] were referenced for the histological description of the structures examined. Additionally, the following histochemical stains were employed to evaluate the composition of glandular secretion: PAS, AB pH 1.0, AB pH 2.5, AB pH 2.5/PAS, and HDI [51–54].

- PAS: Characterizes glycans, glycoconjugates, and neutral glycoproteins.
- AB pH 1.0: Visualizes strongly sulfated mucosubstances.
- AB pH 2.5: Identifies acid sialylated glycosaminoglycans.
- AB pH 2.5/PAS: Identifies sulfated and carboxylated acid mucopolysaccharides, sulfated and carboxylated sialomucins (glycoproteins) (blue color), and neutral mucins (magenta color).
- HDI: Detects the presence of sulfated acid mucosubstances (SAM) and carboxylated acid mucosubstances (CAM).

The histochemical evaluation of the examined structures was described in accordance with the method provided by Spicer and Henson, where (-) indicated a negative reaction; (-/+) and (+) denoted a weak reaction; (++) signified a mild reaction; and (+++) represented a strong reaction [55].

## Results

### The orbital region analysis

The dimensions of the orbit of the studied animals, considering both sides, are presented in Fig. 2a and c; Table 2. In the two examined red pandas, the orbital index and orbital area were larger in the right orbit.

**Table 2:** Dimensions of the orbital parameters. CV% – coefficient of variation; S.D. – standard deviation; n. a. – not applicable; L – left; R – right

Parameters	Side	Mean	S.D.	CV%	Range	Overall			
						Mean	S.D.	CV%	Range
orbital vertical length (mm)	L	18.438	0.11	0.59	18.22–18.73	18.125	0.35	1.95	17.64–18.73
	R	17.812	0.19	1.08	17.64–18.11				
orbital horizontal width (mm)	L	16.546	0.08	0.48	16.44–16.71	16.985	0.48	2.84	16.44–18.14
	R	17.424	0.36	2.07	16.66–18.14				
orbital index (%)	L	89.726	0.74	0.82	89.069–90.669	93.777	4.25	4.53	89.069-102.024
	R	97.828	2.22	2.27	92.452-102.024				
orbital depth (mm)	L	33.589	0.31	0.92	32.96–34.12	34.504	0.96	2.79	32.96–36.41
	R	34.419	0.41	1.16	34.63–36.41				
orbital area (mm^2^)	L	239.635	1.83	0.76	236.429-243.636	243.382	8.72	3.58	234.11-277.353
	R	247.128	11.64	4.71	234.31-277.353				
interorbital distance (mm)	at rostral level	27.676	0.42	1.52	27.16–28.31	n. a.	n. a.	n. a.	n. a.
	at middle level	29.074	0.5	1.71	28.31–29.76	n. a.	n. a.	n. a.	n. a.
	at caudal level	71.543	0.49	0.68	70.99–72.33	n. a.	n. a.	n. a.	n. a.
frontal length (mm)	L	15.225	0.23	0.15	14.87–15.51	15.085	0.23	1.56	14.68–15.51
	R	14.945	0.12	0.82	14.68–15.11				
lacrimal length (mm)	L	11.984	0.28	2.38	11.36–12.33	12.165	0.27	2.29	11.36–12.52
	R	12.346	0.09	0.8	12.25–12.52				
malar length (mm)	L	14.735	0.11	0.77	14.66–14.98	14.082	0.68	4.82	13.22–14.98
	R	13.46	0.15	1.14	13.22–13.56				

The orbit of the examined animals was conically shaped and of an open type. The orbital ring contained an orbital ligament that connected a faintly marked frontal process of the zygomatic bone to a short, sharply ended zygomatic process of the frontal bone (Fig. 2d). The zygomatic arch was thin and prominently arched outwardly. The bones comprising the orbital region included: a broad orbital portion of the frontal bone, a small and narrow facial portion, a large and flat orbital surface of the lacrimal bone, a prominent sphenoidal process of the palatine bone, and a sizable pterygoid process of the basisphenoid bone. The maxillary alveolar ridge expanded posteriorly into a tuberosity of the maxilla (Fig. 2f). Adjacent to this structure in the nasal direction, a vast pterygopalatine surface housed a large maxillary foramen, which transitioned through a short infraorbital canal (approximately 1.7–1.9 cm) into a broad infraorbital foramen at the level of the 2–3 buccal teeth (Fig. 2e and f). Medially from the maxillary tuberosity on the perpendicular plate of the palatine bone, a small canal divided by a transverse bone plate led to two-minute openings: the sphenopalatine foramen and the palatine caudal foramen directly beneath it (Fig. 2g). A prominent thickened protuberance, the caudal lacrimal process, was observed at the orbital margin of the lacrimal bone (Fig. 2h). Red pandas lacked a supraorbital foramen and the fossa for the ventral oblique muscle. An evident supraorbital notch was present on the anterior part of the supraorbital margin, succeeded by a deep trochlear fovea for the superior oblique muscle (Fig. 2h). On the facial surface of the lacrimal bone, a pronounced external lacrimal fossa was visible, while its orbital surface contained a small and less distinct fossa for the lacrimal sac. This fossa led to a single large lacrimal foramen (located directly beneath the maxillary foramen) that connected to the lacrimal canal (Fig. 2g and h). On the temporal surface of the frontal bone, a minimal orbitotemporal crest was observed in the studied animals. Directly below this crest was a solitary small ethmoid foramen (Fig. 2i). Roughly 1.0–1.1 cm from the ethmoid foramen, the optic canal was situated within the wings of the presphenoidal bone (Fig. 2i). Approximately 0.8–0.9 cm from the optic canal, above the marked pterygoid crest, a large orbital fissure was located, with a smaller foramen rotundum immediately below it on the wings of the presphenoid bone (Fig. 2j). Additionally, a medium-sized rostral alar foramen was located on the wings of the presphenoid bone, which, through a brief alar (pterygoid) canal (approximately 0.4–0.5 cm long), led to a medium-sized caudal alar foramen (Fig. 2k).

### The upper and lower eyelids morphology

**Fig. 3:**
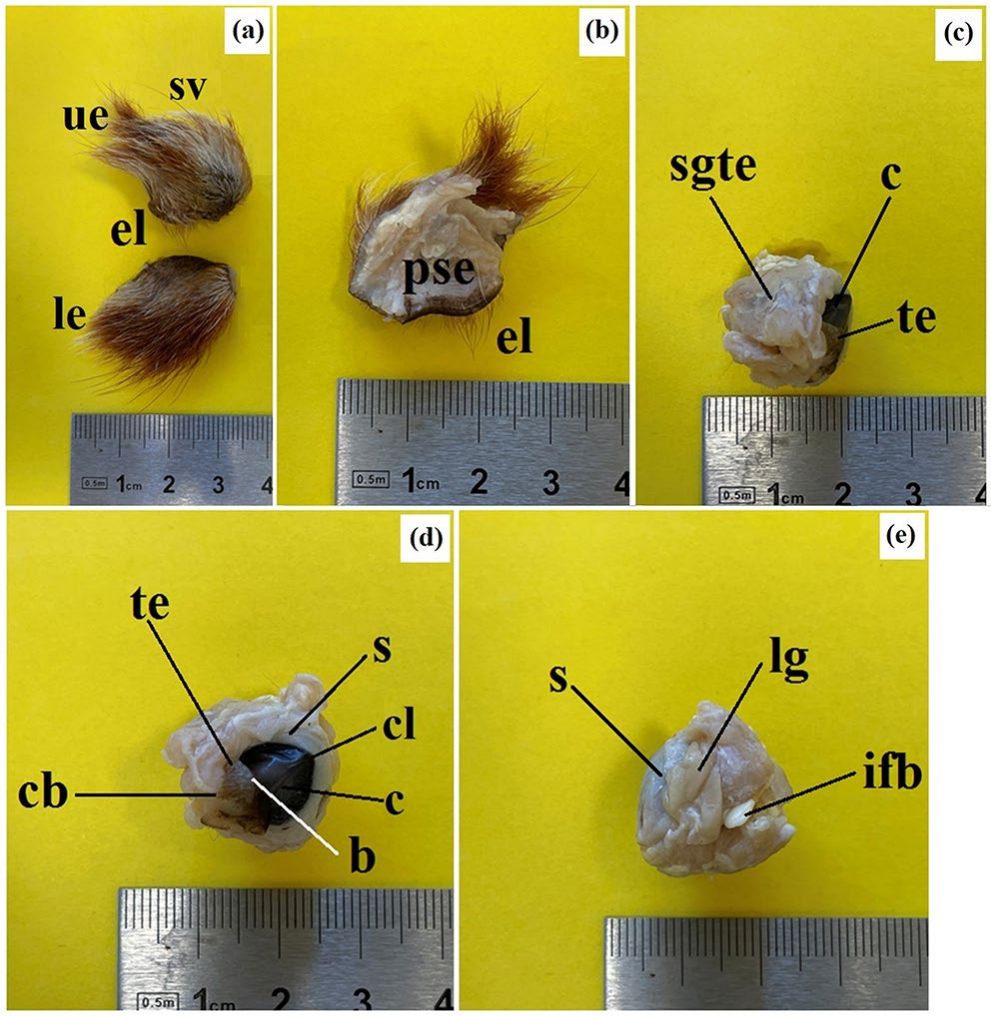
The photomacrograph of the *Ailurus fulgens fulgens* upper and lower eyelids: (**a**) le – lower eyelid, sv – supraorbital vibrissae, ue – upper eyelid; (**b**) el – eyelashes, pse – posterior surface of the eyelid; (**c**) c – cornea, sgte – superficial gland of the third eyelid, te – third eyelid; (**d**) b – branch of the third eyelid (upper and lower), c – cornea, cb – crossbar of the third eyelid, cl – corneal limbus, s – sclera, te – third eyelid; (**e**) ifb – intraperiorbital fat body, lg – lacrimal gland, s – sclera. Scale bars: (**a** – **e**) 4 cm

On the anterior palpebral margin of the UE, soft, medium-length eyelashes were observed, while they were absent on the LE (Fig. 3a). Additionally, 5–6 long sensory hairs (supraorbital vibrissae) were found above the UE. The palpebral conjunctiva was highly pigmented, showing a brown color (Fig. 3b). The morphometric parameters (length, width, thickness) of both the UE and LE from both sides of the examined animals are presented in Table 3. The parameters obtained were comparable between the two male red pandas.

**Table 3:** Dimensions (mm) of the macroscopic parameters of the eyelids and orbital glands. CV% – coefficient of variation; S.D. – standard deviation; L – left; R – right

Parameters	Side	Mean	S.D.	CV%	Range	Overall			
						Mean	S.D.	CV%	Range
upper and lower - length	L	15.189	0.59	3.89	14.26–16.12	15.237	0.55	3.64	14.26–16.12
	R	15.285	0.55	3.63	14.36–16.06				
upper eyelid - width	L	9.385	0.5	5.42	8.66–10.13	8.344	1.16	13.97	6.46–10.13
	R	7.304	0.58	7.98	6.46–8.57				
upper eyelid - thickness	L	2.841	0.17	5.98	2.49–3.11	2.806	0.20	7.19	2.49–3.14
	R	2.770	0.24	8.8	2.57–3.14				
lower eyelid - width	L	8.142	0.21	2.61	7.96–8.74	8.259	0.29	3.57	7.96–9.12
	R	8.376	0.31	3.76	7.88–9.12				
lower eyelid - thickness	L	2.693	0.09	3.49	2.54–2.88	2.644	0.1	3.78	2.74–2.88
	R	2.595	0.08	3.42	2.47–2.78				
superficial gland of the third eyelid - length	L	9.286	0.38	4.18	8.89–10.04	9.614	0.45	4.75	8.89–10.24
	R	9.941	0.25	2.55	9.44–10.24				
superficial gland of the third eyelid - width	L	7.021	0.26	3.78	6.74–7.47	7.111	0.37	5.21	6.26–8.11
	R	7.2	0.41	5.75	6.26–8.11				
superficial gland of the third eyelid - thickness	L	3.711	0.22	6.03	3.36–4.11	3.731	0.21	5.86	3.3–4.11
	R	3.751	0.22	6.05	3.47–4.11				
third eyelid length of crossbar	L	6.886	0.2	2.91	6.61–7.12	6.974	0.18	2.61	6.61–7.14
	R	7.061	0.11	1.67	6.87–7.14				
third eyelid length of upper and lower branch	L	6.443	0.06	0.99	6.36–6.52	6.646	0.25	3.88	6.36–7.11
	R	6.85	0.21	3.19	6.45–7.11				
lacrimal gland - length	L	7.637	0.19	2.55	7.32–7.89	7.771	0.222	2.85	7.32–8.13
	R	7.905	0.18	2.34	7.55–8.13				
lacrimal gland - width	L	3.787	0.22	5.83	3.42–4.12	3.842	0.2	5.38	3.42–4.12
	R	3.896	0.18	2.85	3.58–4.11				
lacrimal gland - thickness	L	2.689	0.14	5.92	2.44–2.95	2.652	0.13	5.24	2.44–2.95
	R	2.616	0.12	4.81	2.47–2.89				

**Fig. 4:**
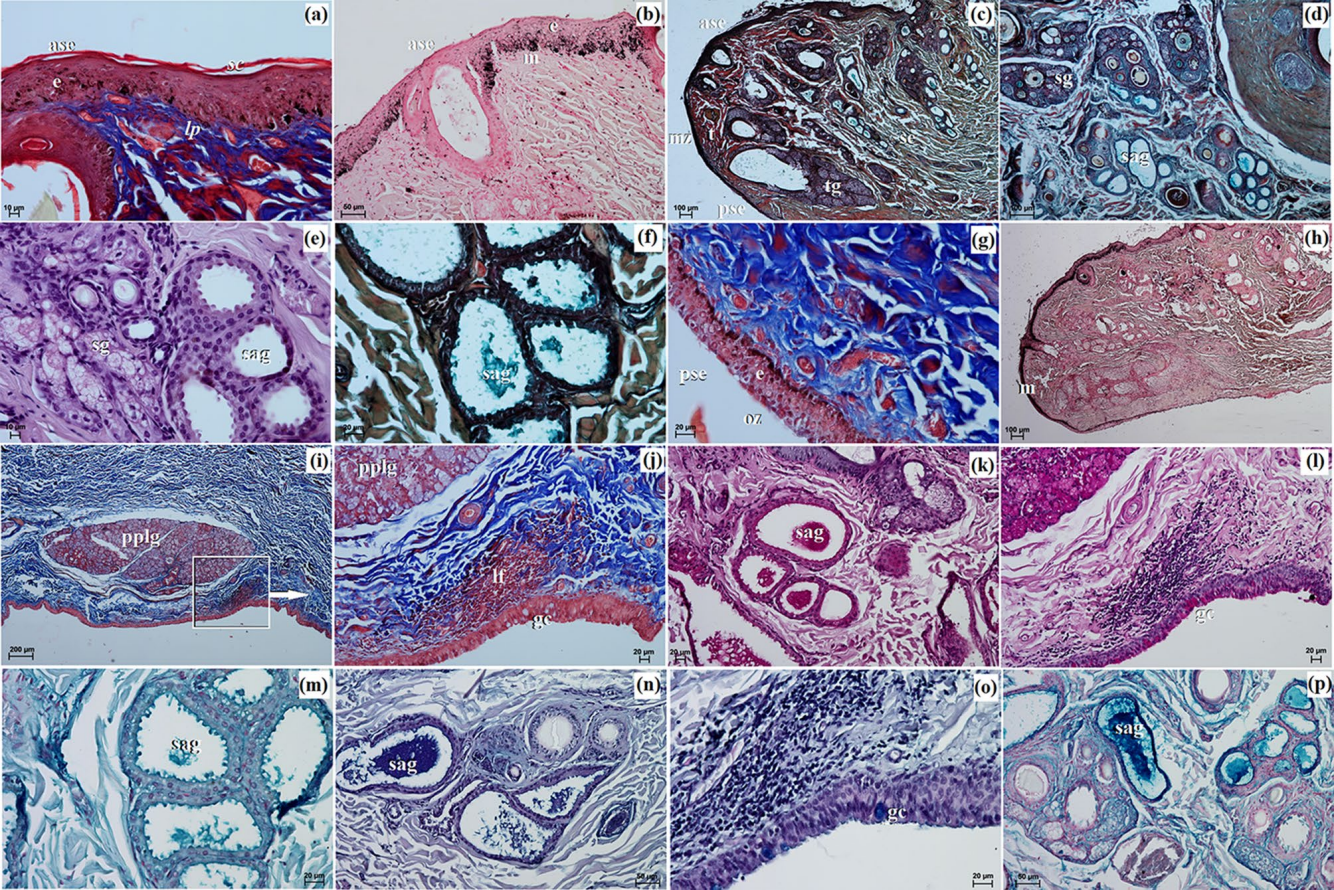
The photomicrograph of the *Ailurus fulgens fulgens* upper and lower eyelids: (**a**) ase – anterior surface of eyelid, e – epithelium, *lp – lamina propria*, ; *sc* – *stratum corneum*; (**b**) ase – anterior surface of eyelid, e – epithelium, m – melanocytes; (**c**) ase – anterior surface of eyelid, mz – marginal zone, pse – posterior surface of eyelid, se – stroma of eyelid, t – tarsus, tg – tarsal gland; (**d**, **e**) sag – simple alveolar gland, sg – sebaceous glands; (**f**, **k**, **m**, **n**, **p**) sag – simple alveolar gland; (**g**) e – epithelium, oz – ocular zone, pse – posterior surface of eyelid; (**h**) m – melanocytes; (**i**) pplg – palpebral part of the lacrimal gland; (**j**) gc – goblet cells, lf – lymphoid follicle, pplg – palpebral part of the lacrimal gland; (**l**, **o**) gc – goblet cells. (**a**, **g**, **i**, **j**) picro-Mallory trichrome stain; (**b**, **h**) Fontana-Masson stain; (**c**, **d**, **f**) Movat-pentachrome stain; (**e**) H&E stain; (**k**, **l**) PAS stain; (**m**) AB pH 2.5 stain; (**n**, **o**) AB pH 2.5/PAS stain; (**p**) HDI stain. Scale bars: (**i**) 200 μm; (**c**, **d**, **h**) 100 μm; (**b**, **n**, **p**) 50 μm; (**f**, **g**, **j**–**m**, **o**) 20 μm; (**a**, **e**) 10 μm

**Fig. 5:**
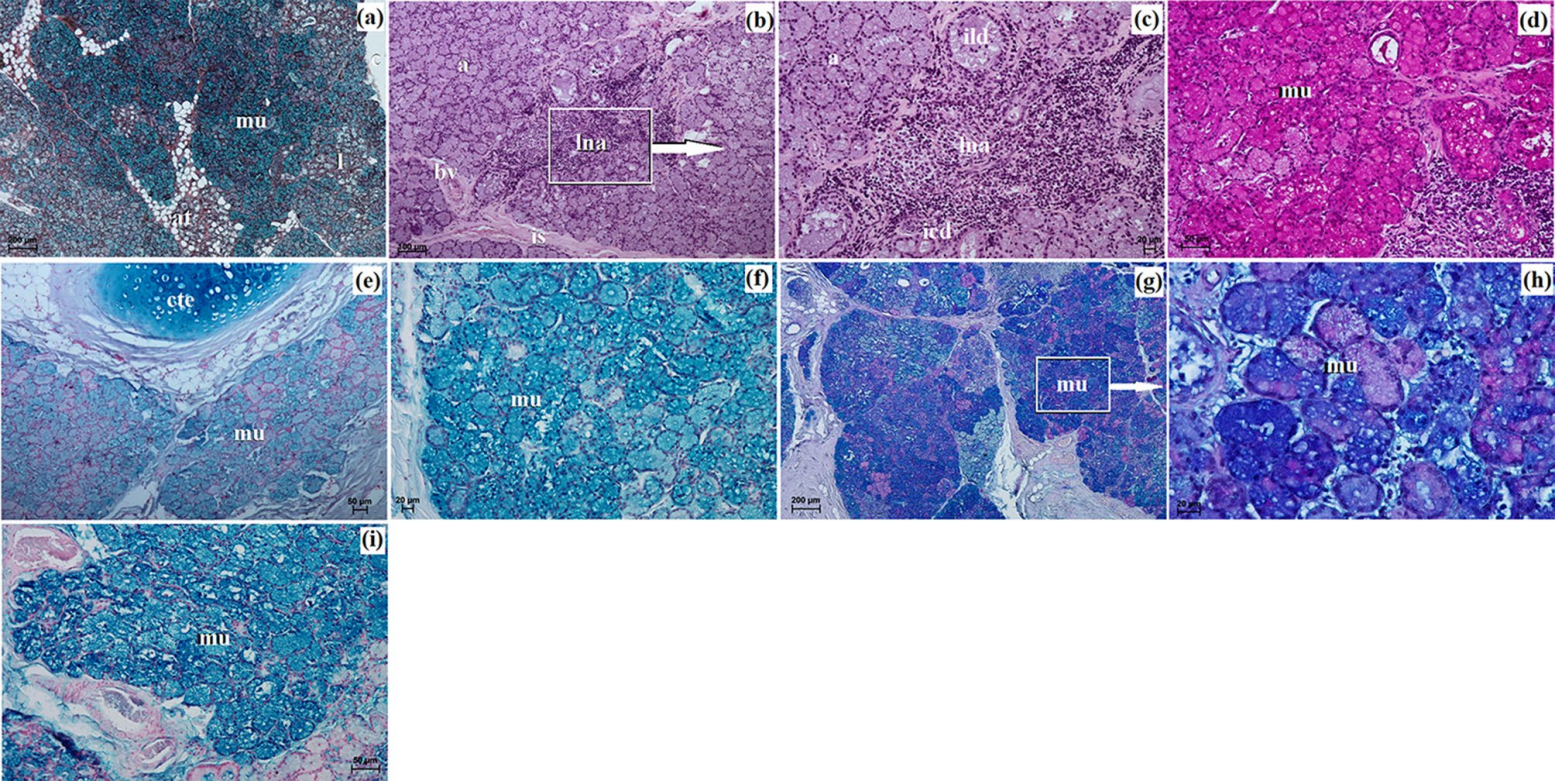
The photomicrograph of the *Ailurus fulgens fulgens* superficial gland of the third eyelid: (**a**) at – adipose tissue, c – capsule, l – lobes, mu – mucous units; (**b**) a –acini, bv – blood vessels, is – interlobar septa, lna – lymph nodule aggregate; (**c**) a –acini, icd – intercalated duct, ild – interlobar duct, lna – lymph nodule aggregate; (**e**) cte – cartilage of the third eyelid, mu – mucous units; (**f** – **i**) mu – mucous units. (**a**) Movat-pentachrome stain; (**b**, **c**) H&E stain; (**d**) PAS stain; (**e**) AB pH 1.0; (**f**) AB pH 2.5 stain; (**g**, **h**) AB pH 2.5/PAS stain; (**i**) HDI stain. Scale bars: (**a**, **g**) 200 μm; (**b**) 100 μm; (**d**, **e**, **i**) 50 μm; (**c**, **f**, **h**) 20 μm

The UE and LE consisted of two parts: the anterior surface and the posterior surface, which included both the marginal zone and the ocular zone. The anterior surface was covered by a stratified squamous epithelium comprising 8 to 15 layers of nucleated cells. The superficial layer of this epithelium was overlaid with a thin *stratum corneum* measuring 7.424 ± 1.01 μm in thickness (Fig. 4a). Within the stratum basale, the epithelial cells, which lay on the basement membrane, contained a significant number of melanocytes (Fig. 4b). Both eyelids displayed well-developed, highly branched tarsal glands and a thick tarsal plate composed of dense fibrous connective tissue (Fig. 4c). The stroma of the eyelids consisted of dense, irregularly arranged fibrous connective tissue containing collagen fibers, distinct reticular fibers, and elastic fibers. This stroma also contained numerous highly branched sebaceous glands and simple alveolar glands (Fig. 4c and e). The simple alveolar glands were composed of a single layer of cubic epithelium and produced a mucinous secretion (Fig. 4f). The posterior surface of the eyelids was divided into two regions: the marginal zone, which constituted the outer border of both eyelids, and the ocular zone, which encompassed the conjunctival area in contact with the eyeball (Fig. 4c and g). The marginal zone was lined with a stratified columnar epithelium containing 7 to 9 layers of nucleated cells. The epithelial cells within the stratum basale of this zone showed prominently marked melanin granules (Fig. 4h). The ocular zone was covered by 6 to 8 layers of non-keratinized cells (Fig. 5i and j). In the UE, the palpebral part of the LG was present (Fig. 4i and j). Additionally, lymphoid follicles were observed only in the UE (Fig. 4j).

The histochemical analysis revealed a negative reaction in the tarsal and sebaceous glands and a moderate to strong reaction in the simple alveolar glands with proteinaceous staining. Goblet cells exhibited moderate to strong staining across all stains. The results of the histochemical staining are summarized in Table 4; Fig. 4k and p.

**Table 4:** Histochemical analysis of the structure of the upper and lower eyelids, superficial gland of the third eyelid with third eyelid and lacrimal gland. PAS – periodic Acid-Schiff; AB pH 1.0 – alcian blue pH 1.0; AB pH 2.5 – alcian blue pH 2.5; HDI – Hale’s dialyzed iron stain

Structure Methods	Simple alveolar glands	Sebaceous glands	Tarsal glands	Goblet cells	Superficial gland of the third eyelid	Third eyelid (goblet cells)	Lacrimal gland
PAS	++	–	–	++	+++	+++	++/+++
**AB pH 1.0**	+	–	–	+	+	+	+ (dominant) and ++ (sparse)
**AB pH 2.5**	+++	–	–	+++	++ (sparse) and +++ (dominant)	+++	++/+++
**AB pH 2.5/PAS**	+/++ (blue color)	–	–	++ (blue color)	+/++ (sparse magenta color) and +++ (dominant blue color)	++ (blue color)	+/++ (magenta color) and ++/+++ (blue color)
**HDI**	+/++	–	–	+/++	++/+++	++	+/++

### The orbital glands’ morphology

The morphometric parameters (length, width, thickness) of the SGTE, the TE itself, and the LG in the two male red pandas are presented in Table 3. The results obtained were consistent.

The SGTE was oval and light pink in color (Fig. 3c). It was situated in the medial angle of the eye, between the medial straight and ventral straight muscles of the eyeball and was partially covered by the ventral oblique muscle of the eyeball.

This gland was surrounded by a large intraperiorbital fat body, beneath which lay a very thick connective tissue capsule (Fig. 5a). Thin interlobar septa extended from the connective tissue capsule deep into the gland, with numerous clusters of fat cells located laterally. These interlobar septa divided the gland structure into large, medium, and small lobes (Fig. 5a). The connective tissue capsule consisted of collagen and elastic fibers, as well as arteries, veins, and nerves. Histological analysis revealed it to be a multilobar acinar complex gland producing a mucous secretion (Fig. 5a). The acini were characterized by a small lumen lined with tall conical cells exhibiting eosinophilic cytoplasm (Fig. 5b and c). The Movat-pentachrome stain indicated the presence of mucous secretory units with a strong reaction (+++) (Fig. 5a). The intercalated ducts were lined with cuboidal epithelium. Within the central part of the lobes and the interlobar septa, numerous interlobular ducts were observed, composed of a simple columnar epithelium (Fig. 5c). Lymph nodules aggregated within the interlobular ducts (Fig. 5b and c).

Histochemical analysis revealed strong staining results in the acini across all staining methods. The results of the histochemical staining are summarized in Fig. 5d and i; Table 4.

### Third eyelid

The marginal part of the TE was highly pigmented (brown-black color) and thin. The TE had a T-shaped structure, consisting of an upper and lower branch and a crossbar, which was flanked by the SGTE (Fig. 3d). It was situated in the medial angle of the eyeball.

**Fig. 6:**
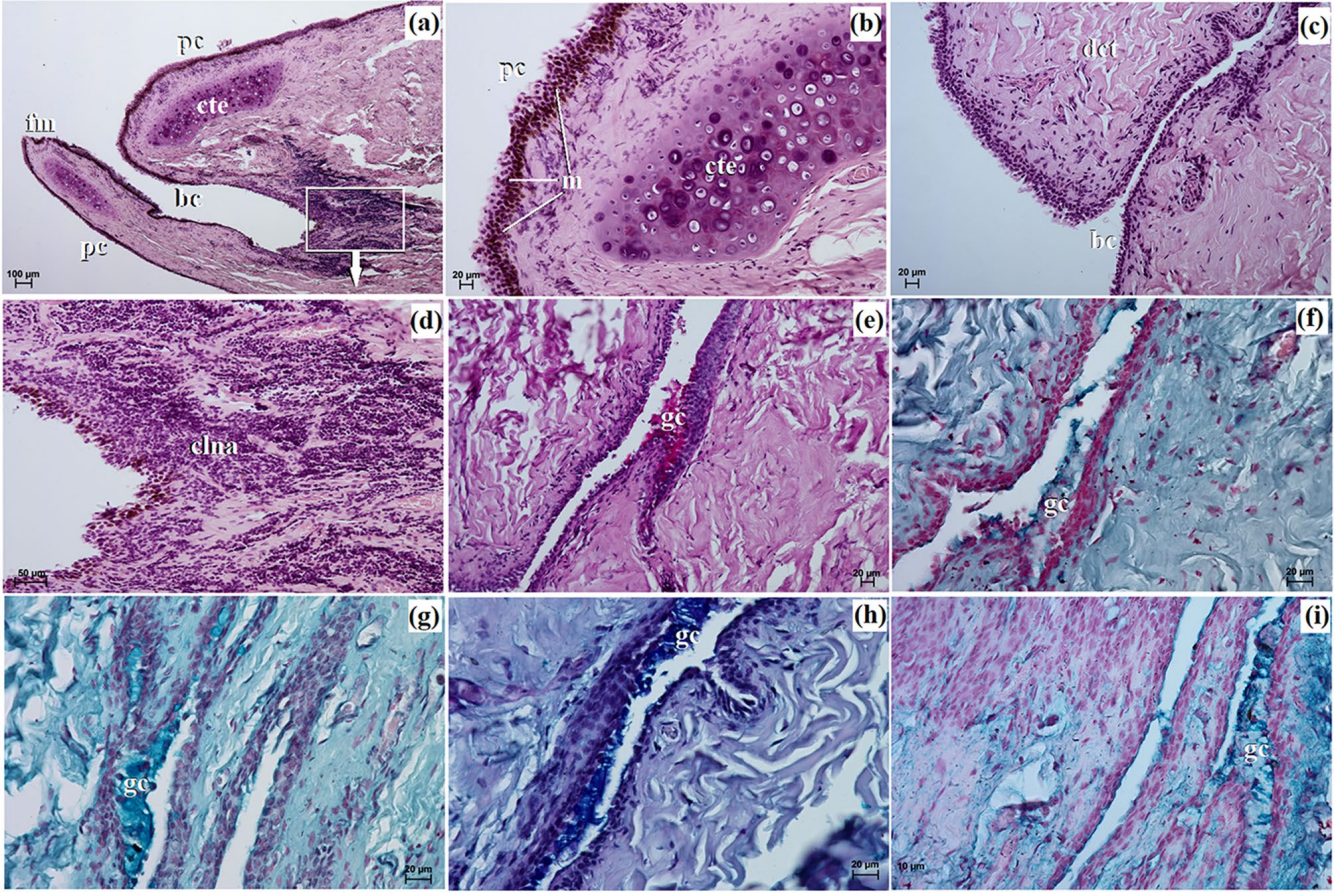
The photomicrograph of the *Ailurus fulgens fulgens* third eyelid: (**a**) bc – bulbar conjunctiva, cte – cartilage of the third eyelid, fm – free margin, pc – palpebral conjunctiva; (**b**) cte – cartilage of the third eyelid, m – melanocytes, pc – palpebral conjunctiva; (**c**) bc – bulbar conjunctiva, dct – dense connective tissue; (**d**) clna – conjunctival lymphoid nodule aggregate; (**e** - **i**) gc – goblet cells. (**a** – **d**) H&E stain; (**e**) PAS stain; (**f**) AB pH 1.0; (**g**) AB pH 2.5 stain; (**h**) AB pH 2.5/PAS stain; (**i**) HDI stain. Scale bars: (**a**) 100 μm; (**d**) 50 μm; (**b**, **c**, **e** – **h**) 20 μm; (**i**) 10 μm

Histologically, the palpebral conjunctiva of the TE was covered by a cubic multilayer epithelium composed of 4 to 5 layers of nucleated cells, while the bulbar conjunctiva contained a cubic multilayer epithelium consisting of 10 to 13 layers of epithelial cells (Fig. 6a and c). The free margin of the TE contained numerous melanocytes and was composed of irregularly woven fibrous connective tissue with a few blood vessels. The surface cartilage was enveloped by thick layers of perichondrium (comprising collagen and elastic fibers, fibroblasts, and blood vessels) and numerous adipocytes. The cartilage of the TE was made up of hyaline tissue (Fig. 6b). Within the bulbar conjunctiva, a subepithelial tissue conjunctival lymph nodule aggregate was observed (Fig. 6a and d).

Histochemical analysis revealed a strongly positive reaction in PAS, AB pH 2.5, AB pH 2.5/PAS, and HDI staining in the goblet cells, except for AB pH 1.0 where only a slight reaction was noted. The results of the histochemical staining of the goblet cells are summarized in Fig. 6e and i; Table 4.

### The lacrimal gland

The LG was approximately triangular to oval in shape and light pink in color (Fig. 3e). It was situated in the lateral angle of the eye, between the dorsal straight and lateral straight muscles of the eyeball, at the dorsolateral angle of the periorbita.

**Fig. 7:**
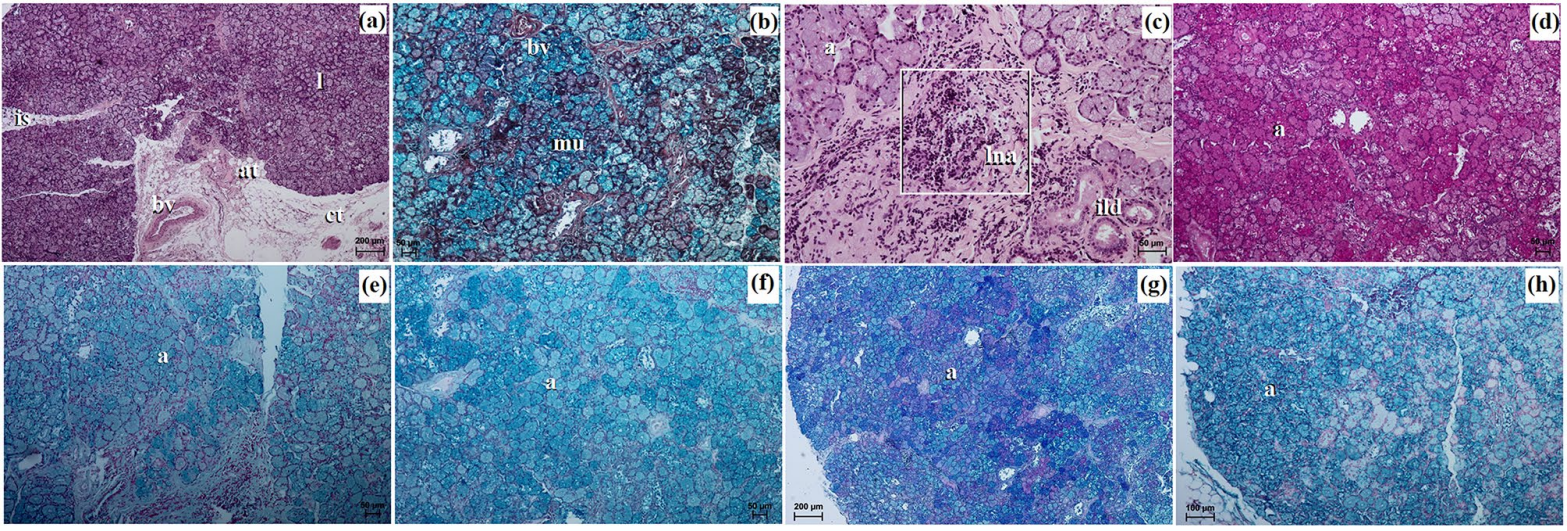
The photomicrograph of the *Ailurus fulgens fulgens* lacrimal gland: (**a**) at – adipose tissue, bv – blood vessels, c – capsule, ct – connective tissue, is – interlobar septa, l – lobes; (**b**) bv – blood vessels, mu – mucous units; (**c**) a – acini, ild – interlobar duct, lna –lymph nodule aggregate; (**d** - **h**) a – acini. (**a**, **c**) H&E stain; (**b**) Movat-pentachrome stain; (**d**) PAS stain; (**e**) AB pH 1.0; (**f**) AB pH 2.5 stain; (**g**) AB pH 2.5/PAS stain; (**h**) HDI stain. Scale bars: (**a**, **g**) 200 μm; (h) 100 μm; (**b** – **f**) 50 μm

The LG revealed a multilobar acinar complex structure producing a mucous secretion (Fig. 7a and b). It was surrounded by a thin connective tissue capsule, covered with numerous clusters of adipose cells that formed indistinct connective tissue septa, dividing the gland into very large lobes (Fig. 7a). This connective tissue consisted of collagen fibers, reticular and elastic fibers, blood vessels, and nerves. The acini featured a small lumen and were composed of tall conical secretory cells with eosinophilic cytoplasm (Fig. 7c). Numerous intercalated ducts were lined with a monolayer of cubic epithelium. Movat pentachrome staining indicated the presence of mucous units with a strongly positive reaction (+++), confirming the mucous nature of the LG secretion (Fig. 7b). The presence of a lymphoid follicle surrounding the interlobular ducts was noted (Fig. 7c).

Histochemical analysis revealed predominantly moderate positive staining reactions in the acini. The results of the histochemical staining are summarized in Fig. 7d and h; Table 4.

### The eyeball morphometry and eye tunics

The dimensions of the eyeballs in the two male red pandas are presented in Table 3. Morphometric analysis of the eyeball, both left and right, revealed that these parameters were comparable.

**Fig. 8:**
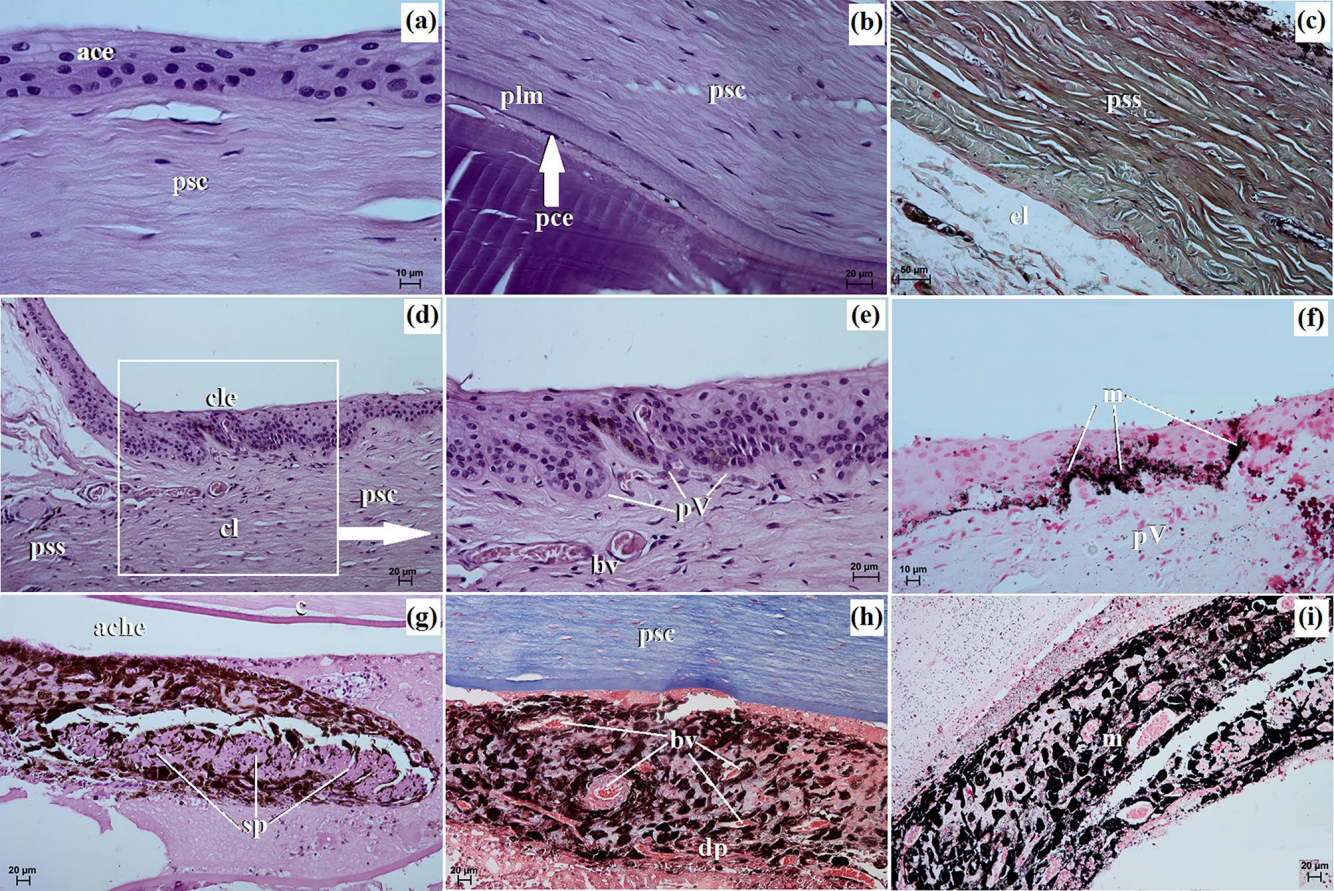
The photomicrograph of the *Ailurus fulgens fulgens* eyeball tunics. (**a**) Cornea: ace – anterior corneal epithelium, psc – proper substance of cornea; (**b**) Cornea: pce – posterior corneal epithelium (white arrow), plm – posterior limiting membrane, psc – proper substance of cornea; (**c**) Sclera: el – episcleral lamina, pss – proper substance of sclera; (**d**) cl – corneal limbus, cle – corneal limbus epithelium, psc – proper substance of cornea; (**e**) bv – blood vessels, pV – palisades of Vogt; (**f**) Iris with sphincter muscle: m – melanocytes, pV – palisades of Vogt; (**g**) Iris with sphincter muscle: ache – anterior chamber of the eyeball, c – cornea, sp – sphincter pupil; (**h**) Iris with the small diameter blood vessels: bv – blood vessels, dp – dilator of pupil, psc – proper substance of cornea; (**i**) Iris with dominant melanocytes: m – melanocytes. (**a**, **b**, **d**, **e**, **g**) H&E stain; (**c**) Movat-pentachrome stain; (**f**, **i**) Fontana-Masson stain; (**g**, **h**) picro-Mallory trichrome stain. Scale bars: (**c**) 50 μm; (**b**, **d**, **e**, **g** – **i**) 20 μm; (**a**, **f**) 10 μm

The cornea comprised four layers, starting from the outer surface and progressing inward to the anterior corneal epithelium, which was a non-cornified stratified squamous epithelium consisting of 6 to 8 layers of cells. The basal layer of this epithelium consisted of 1–2 layers of cubic cells with large oval nuclei. The thickness of the anterior corneal epithelium was measured at 32.541 ± 3.6 μm. The second layer was the proper substance of the cornea, composed of dense fibrous connective tissue consisting of collagen fibers and numerous flattened keratocytes. The thickness of the proper substance of the cornea was 813.244 ± 9.8 μm. The third layer was the posterior limiting membrane, known as Descemet’s membrane, which consisted of regularly arranged collagen fibers and showed a strongly positive (+++) PAS reaction. The thickness of the posterior limiting membrane was 16.686 ± 1.2 μm. The fourth layer was the posterior corneal epithelium, which was composed of a single-layer squamous epithelium (Fig. 8a and b). The anterior limiting membrane, known as Bowman’s membrane, was not identified in the examined animals.

The sclera was primarily composed of dominant collagen fibers arranged in crisscrossing bundles, along with a network of delicately marked elastic fibers. Additionally, numerous blood vessels, fibroblasts, and clusters of melanin granules constituted the proper substance of the sclera. On the outer surface of the sclera, there was a layer of loose fibrous connective tissue, comprising collagen and elastic fibers, and to a lesser extent, reticular fibers. This layer also contained fibrocytes, fibroblasts, histiocytes, and mast cells, forming the episcleral lamina. The dark lamina of the sclera was prominent due to the abundant presence of melanin granules, fibroblasts, and blood vessels (Fig. 8c).

The corneal limbus was located at the border between the sclera and cornea. Within the corneal limbus epithelium, the palisades of Vogt were present at the junction of the cornea and sclera. The corneal limbus epithelium consisted of 7 to 10 layers of epithelial cells: 1–2 layers of flattened superficial cells with a squamous nucleus, 5–6 layers of intermediate wing cells with an oval nucleus, and 1–2 layers of basal cells with round nuclei. Melanin granules were found in all cell layers (Fig. 8d and f).

**Fig. 9:**
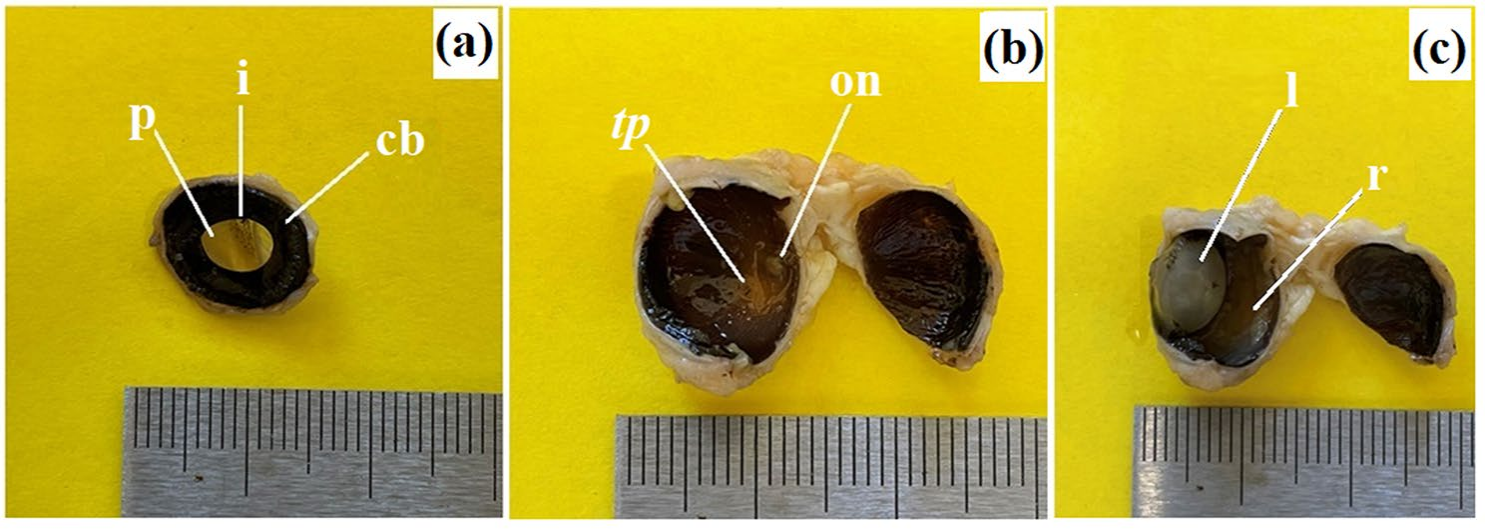
The photomacrograph of the *Ailurus fulgens fulgens* structures of the eyeball: (**a**). cb – ciliary body, i – iris, p – pupil; (**b**) on – optic nerve, *tp* – *tapetum lucidum*; (**c**) l – lens,, r – retina. Scale bars: (**a** – **c**) 3 cm

The pupil of these pandas was horizontally ovoid in its resting (*post-mortem*) state (Fig. 9b).

**Fig. 10:**
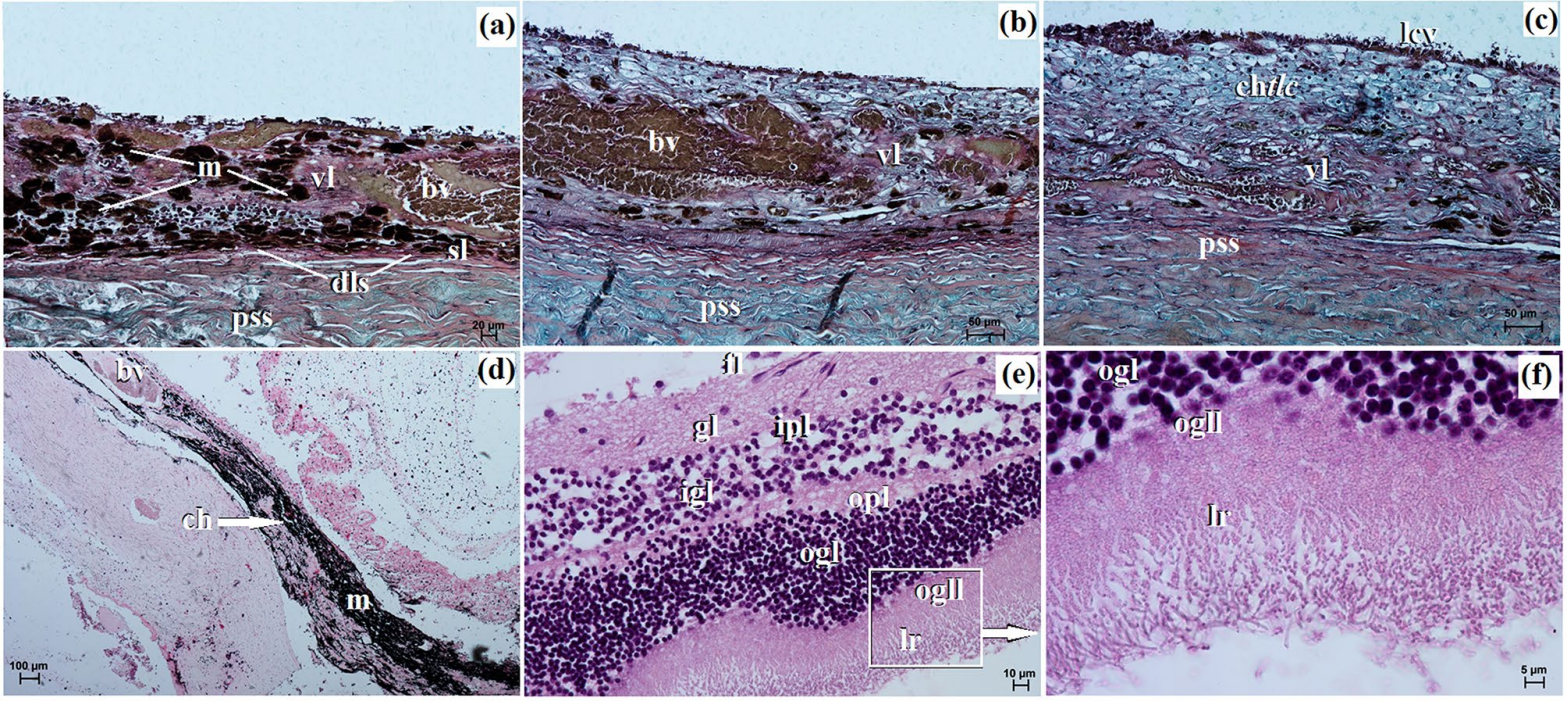
The photomicrograph of the *Ailurus fulgens fulgens* eyeball tunics. (**a**) Choroid: bv – blood vessels, dls – dark lamina of sclera, m – melanocytes, pss – proper substance of sclera, sl – suprachoroid layer, vl – vascular layer; (**b**) Choroid with vascular layer: bv – blood vessels, pss – proper substance of sclera, vl – vascular layer; (**c**) Choroid with choroidal *tapetum lucidum cellulosum*: ch*tlc* – choroidal *tapetum lucidum cellulosum*, lcv – lamina of capillary vessels, pss – proper substance of sclera, vl – vascular layer; (**d**) Choroid with dominant melanocytes: bv – blood vessels, ch – choroid, m – melanocytes; (**e**) Retina: fl. – a fiber layer of the optic nerve, gl – a ganglion cell layer, igl – an inner granular layer, ipl – an inner plexiform layer, ogl – an outer granular layer, ogll – an outer glial limiting layer, opl – an outer plexiform layer; (**f**) Retina: ogl – an outer granular layer, ogll – an outer glial limiting layer, opl – an outer plexiform layer; lr – a layer of rods. (**e**, **f**) H&E stain; (**a** –**c**) Movat-pentachrome stain; (**d**) Fontana-Masson stain. Scale bars: (**d**) 100 μm; (**c**, **b**) 50 μm; (**a**) 20 μm; (**e**) 10 μm; (**f**) 5 μm

The iris presented a black-brown color (Fig. 10a) and was comprised of three parts: the anterior iris epithelium, which consisted of a single-layer squamous epithelium; the outer limiting layer, composed of collagen fibers interspersed with fibrocytes and melanin granules; and the stroma of the iris. The stroma contained very small diameter blood vessels (55.314 ± 20.9 μm), fine collagen fibers, fibroblasts, macrophages containing phagocytosed melanin, which formed numerous clusters filling almost the entire iris stroma, as well as nerves and two smooth muscles: a well-developed sphincter of pupil and dilator of pupil (Fig. 8g and i). On the posterior surface of the iris, there was a retinal pigment layer consisting of two layers of cells containing melanin granules.

Macroscopically, the ciliary body appeared large and had a round shape (Fig. 9a) and the *tapetum lucidum* appeared as a milky, non-opalescent crescent (Fig. 9b). The thickness of the *tapetum lucidum* ranged from 70.175 ± 19.2 μm to 118.082 ± 7.8 μm.

The lens was a biconvex, round body (Fig. 9c). It was surrounded by a thin capsule measuring 7.7 ± 0.7 μm, which exhibited a strongly positive (+++) PAS reaction.

The choroid consisted of five layers: the suprachoroid layer, composed of dense fibrous connective tissue with densely packed clusters of melanocytes; the vascular layer, composed of blood vessels of very large diameter. Between these vessels, there was loose fibrous connective tissue with poorly defined collagen and elastic fibers, along with numerous clusters of melanin granules; the choroidal *tapetum lucidum cellulosum*, which consisted of loosely packed oval cells ranging from 23.513 ± 4.0 μm to 32.9 ± 3.7 μm in diameter with large round nuclei (Fig. 10a and c). These cells were irregularly arranged in 5 to 9 layers, surrounded by fine collagen fibers and numerous clusters of melanin granules (Fig. 10d). No capillary vessels were observed within this *tapetum lucidum*; the lamina of capillary vessels and the basal layer (Bruch’s membrane) were composed of capillary endothelial cells, fine collagen and elastic fibers, along with the basal membrane of the retinal pigment layer (Fig. 10a and c). The thickness of the choroid was 244.915 ± 36.8 μm. It was not possible to visualize the ciliary body in the examined histological preparations.

The retina’s optic part showcased a complex structure with ten neatly stacked layers. These layers, in order, began with the pigment layer that directly touches the choroid. Following this were: a pigment epithelium layer; a layer dominated by rods and cones; an outer glial limiting layer, an outer granular layer; an outer plexiform layer, an inner granular layer, an inner plexiform layer, a ganglion cell layer; a fibre layer leading to the optic nerve and an internal glial limiting layer (Fig. 10e and f).

The examined red panda has an oval optic disc, and the color fundus was milky white in which traces of delicate blood vessels were visible (Fig. 9b). The diameter of the optic disc in the examined pandas was 1.511 ± 0.07 mm.

## Discussion

The red panda is the only member of the unique Ailuridae family and holds an uncertain phylogenetic position [4]. From its inception, determining the appropriate placement of the red panda on the evolutionary tree has been a subject of considerable controversy and challenge. Initial research suggested that the red panda belonged to the Procyonidae family (specifically the subfamily Ailurinae, as it was thought to be related to the giant panda). However, a comparative study in 1982 classified the red panda within the Ailuridae family [10]. Mitochondrial DNA studies conducted by Pecon-Slattery and O’Brien in 1995 indicated a close relationship between the red panda and procyonids [56]. Ultimately, after extensive genetic research, the red panda was assigned to the clade Musteloidea, which also encompasses the Procyonidae, Mustelidae, and Mephitidae families [57]. Regrettably, there is limited research focusing on the orbital region, eye anatomy, or veterinary ophthalmology within the Ailuridae, Procyonidae, Musteloidea, or Mephitidae families [58, 59]. We hope that the research findings presented here will significantly enhance our understanding of comparative anatomy in the orbit, eye tunics, and selected accessory organs of the eye, given that the red panda is the sole living representative of the Ailuridae family.

A detailed anatomical description of the orbital region in the examined red panda reveals that its orbits are of an open type, similar to those of Canidae, Ursidae, and the Musteloidea clade. This orbit is composed of similar bony structures, although differences among the aforementioned groups were also observed [46, 60–66]. Common features shared by Canidae, Ursidae, and the Musteloidea clade include the absence of a supraorbital foramen and the presence of an infraorbital foramen [62–66]. Significant differences exist not only between Canidae and Ursidae but also within these families and the Musteloidea clade itself. Features common to Ailuridae, Canidae, and Ursidae include the presence of an optic canal, orbital fissure, foramen rotundum, and rostral and caudal alar foramen with an alar canal [46, 60–62, 67, 68]. The differences observed between Canidae, Ursidae, and Ailuridae include a single ethmoid foramen in the red panda, Asiatic black bear, and South African painted dog, whereas domestic dog has a double ethmoid foramen. Additionally, fossa for the lacrimal sac is present in red pandas studied by us, as well as in Asiatic black bear, and domestic dog but absent in South African painted dog [46, 60–62, 66]. A shared feature among Ailuridae, Canidae, and Musteloidea clade is the presence of a narrow, short, and pointed zygomatic process of the frontal bone. Unique to the Ailuridae family are the trochlear fovea for the superior oblique muscle, caudal lacrimal process, external lacrimal fossa, and a notably weak orbitotemporal crest. Glatston [1], Groves [10], and Hu et al. [3] report that the Chinese subspecies of the red panda is characterized by a significantly larger zygomatic arch, evident in lateral projection. This finding aligns with our observations for the Himalayan subspecies of the red panda, despite 80–95% of these carnivores primarily consuming bamboo. Dumont et al. [37] conducted a study on skull shape evolution in musteloid carnivorans, considering locomotor habits, diet, and activity. Their research indicated that the zygomatic arch is dorsally curved in carnivorous species, whereas it appears straighter in herbivorous and omnivorous species [35]. The temporal process of the zygomatic bone, which is part of the zygomatic arch, is broader and cranio-ventrally oriented in herbivorous species compared to carnivorous and omnivorous species, where it is less developed. Casares-Hidalgo et al. [69] have suggested that there is no clear association between orbit orientation and the ecology of living carnivorans. These authors hypothesize that the evolution of the orbit in mammalian carnivores represents a unique case of an ecological bottleneck specific to carnivorans. Given that our morphometric study of the orbit involved only two adult males and lacked comparison with adult females, these results should be considered preliminary. Future studies should aim to determine whether sexual dimorphism exists and whether significant differences in orbit dimensions are present between the Himalayan and Chinese subspecies of the Ailuridae family.

Because the macroscopic measurements of selected accessory organs of the eye were conducted on only two male specimens, our findings are also preliminary. Due to the absence of females and the limited number of subjects in this study, we cannot definitively determine the influence of sexual dimorphism on these dimensions. Macroscopic studies of both eyelids in two male red pandas revealed the presence of eyelashes exclusively on the UE, a pattern shared with domestic dog, wild dogs (such as the South African painted dog and crab-eating fox), ring-tailed coati, and the Asiatic black bear [60–62, 70, 71]. Carvalho et al. [70, 72] noted that both the maned wolf and the crab-eating fox exhibit short and sparse accessory eyelashes on the LE, whereas ring-tailed coatis possess a hairy caruncle. The palpebral conjunctiva in the red panda was highly pigmented. According to Carvalho et al. [70], the palpebral conjunctiva in ring-tailed coati displays greater pigmentation in adults than in juveniles. This pigment is translucent and more concentrated near the palpebral limbus [70]. Histologically, the eyelids of the red panda were structurally similar to those of Canidae, Ursidae, and domestic ferret (*Mustela putorius furo*). However, our examined animals exhibited characteristic simple alveolar glands [60–62, 72, 73]. Another distinctive feature observed in the red panda was the presence of the palpebral part of the LG in the UE. This has not yet been demonstrated in other terrestrial Caniformia but is found in humans and aardvarks [60, 74]. Our research also revealed the presence of conjunctiva-associated lymphoid tissue (CALT) in the UE, manifested as lymphoid follicles and diffuse lymphocytes, a pattern similar to that observed in Canidae and Ursidae [60–62, 72–75]. Wenzel-Hora et al. [76] suggested that in domestic dogs, the number, size, and localization of lymphoid cells may be influenced by the age of the animals as well as antigenic stimulation.

Morphometric studies showed that the SGTE red panda, was large. Because our study included only two males, we cannot comment on whether sexual dimorphism may affect the dimensions of the SGTE. The SGTE, like in wild and domestic Canidae and Ursidae, was located in the medial angle of the eye [60–62, 71, 77, 78]. Similarly to the South African painted dog and Asiatic black bear, the SGTE was oval in shape and light pink in color [60, 61]. In contrast, in domestic dogs, it was teardrop-shaped and pink [44].

Histological analysis of the red panda’s SGTE revealed that it is a branched complex alveolar gland that produces mucous secretion. In contrast, the glands in Canidae and Ursidae have a tubuloacinar structure, producing serous-mucous secretion in domestic dog, and serous secretion in the South African painted dog [44, 60, 61, 77].

The TE in the red panda was T-shaped, highly pigmented and located in the medial angle of the eye, consistent with previous in the domestic and wild Canidae, Ursidae and Procyonidae [44, 60–62, 71, 72, 77–82]. According to Johnson [79], in the kinkajou, the TE is well-developed and forms a loose conjunctival sac that contracts over the cornea, whereas in the raccoon, it is vestigial. The histological study on the examined red pandas, as well as domestic and wild Canidae and Ursidae, revealed that the cartilage of the TE consisted of hyaline tissue [60–62, 71, 72]. Our research also demonstrated the presence of conjunctival lymphoid follicle of the TE, consistent with findings in Asiatic black bear, crab-eating fox, domestic dog, and South African painted dog [60–62, 71]. Hermanson et al. [44] reported that the presence of lymphoid follicles in the TE of Canidae is a physiological condition. The follicles are part of the immune system that responds to viral infections, such as Canine herpesvirus-1 (CHV − 1), leading to follicular inflammation of the TE. Additionally, they respond to the presence of foreign bodies in the conjunctival sac and to antigens in the dog’s environment, such as dust or pollen [44, 73].

In our study of the panda, similar to findings in Canidae and Ursidae, we did not observe the presence of the deep gland of the TE, commonly referred to as the Harderian gland. Paule [83] noted that this gland is only found in the ranch mink (*Mustela vison*) and the Eastern raccoon (*Procyon lotor lotor*) among Carnivora. According to Paule [83], this is a small orbital gland that produces a mixed secretion.

Morphometric analysis revealed that the LG in red pandas was smaller compared to the SGTE in the same species and when compared to Canidae [61, 62]. El-naseery et al. [84]. , Lantyer-Araujo et al. [62], Park et al. [78], and Saito et al. [85] demonstrated in their studies on domestic dogs (mongrel dogs and purebred dogs) that sex, breed, and skull type (brachy-, dolicho- and mesocephalic) can influence variations in the dimensions of the LG. The LG in red panda was located in the dorsolateral angle of the eye, a location consistent with findings in Canidae and Ursidae [60–62, 70, 71, 73, 79, 84, 86–106]. In examined animals, these glands exhibited a triangular shape that approached oval. In contrast, the LG in Asiatic black bear was distinctly triangular [60]. As noted by El-naseery et al. [84]. , Park et al. [78], and Zwingenberger et al. [106], the shape of the gland in domestic dogs can vary, even among different breeds. Histological and histochemical analyses revealed that the gland in the red panda presented an acinar branched complex structure with a mucous nature, while the Asiatic black bear displayed a muco-serous character. Whereas in the Canidae family the LG was characterized by a tubuloacinar structure and produced a serous secretion.

The obtained results of the eyeball macroscopic measurements performed in the examined red pandas were similar to tests carried out in the same species (*n* = 1) by Kirk [107], although in our subjects’ dimensions related to the diameter of the cornea, equator eye diameter and axial eye diameter were slightly higher. The dimensions of the eyeball in examined individuals were similar to those of the ringtail (*Bassariscus astutus*), common raccoon, and crab-eating raccoon (*Procyon cancrivorus*) [107, 108]. Some species within Mustelidae family are characterized by smaller and medium-sized eyeballs compared to those of Ailuridae [109]. Heard-Booth & Kirk [110] reported that the size of vertebrate eyes depends on factors such as body size, head size, diet, daily activity, as well as the animal’s speed, visual acuity, and ability to avoid collisions with obstacles in its path [60, 111, 112]. Land and Nilsson [113] and Walls [111] noted that large eyes have longer focal lengths, allowing for the projection of a larger image onto the optic part of the retina. A larger retinal image is sampled by a greater number of photoreceptors than a smaller image, which can lead to increased visual acuity if the density of photoreceptors and ganglion cells, as well as retinal summation, remain constant [110]. Land and Nilsson [113] and Walls [111], based on their experimental studies, demonstrated that, according to Leuckart’s law, the absolute size of the eyeball is significantly positively correlated with the maximum speed of movement of animals. Furthermore, these authors concluded from their research that the maximum speed of movement in animals is one of several crucial selection factors that could have influenced the evolution of eyeball dimensions in animals [110].

Upon analyzing our results of eyeball measurements in the red panda and comparing them with the dimensions of eyeballs in examined species of mammals from the clade Musteloidea, we found that the size of the eyeball is quite similar between the families Ailuridae and Procyonidae. Kirk [107] noted that the activity pattern also plays a significant role in determining eyeball size. For instance, nocturnal animals like those from the genera *Arctonyx, Procyon, Bassariscus*, and *Ailurus* within the clade Musteloidea have relatively larger and more curved eyeballs. This adaptation allows for the collection of maximum light, resulting in brighter retinal images, higher ratios of photoreceptors to ganglion cells, and increased ratios of light-sensitive rods to cones. Conversely, diurnal animals within the clade Musteloidea, such as the yellow-throated marten (*Martes flavigula*), exhibit reduced eyeball size, enhanced visual acuity for resolving fine spatial details through reduced retinal summation, and a flatter curvature for larger retinal images, along with increased cones to rods. Intermediate eyeball sizes are observed in cathemeral animals, specifically within the subfamily Mustelinae.

Histological studies revealed that the cornea in the red panda consists of only four layers, lacking a Bowman’s membrane, similar to findings in the domestic dog, dingo (*Canis dingo*), corsac fox (*Vulpes corsac*), and Asiatic black bear [60, 114, 115]. In our red pandas, as well as in other wild canids (wolf, South African painted dog, crab-eating fox, dingo, and corsac fox), the anterior corneal epithelium is characterized by varying numbers of nucleated cells [61, 114–116]. Gwin et al. [117] and Nautscher et al. [115] reported that the number of nucleated cells in the anterior corneal epithelium correlates with corneal thickness, which itself changes with the age of the individual.

The corneal limbus in the red panda revealed the presence of palisades of Vogt within limbal crypts in the corneal limbal epithelium, similarly, as described in South African painted dog, Asiatic black bear, domestic dog, cat, pig, rat, and human, [60, 61, 86, 87, 115, 118–120]. These palisades consist of conjunctival folds and contain niches for limbal epithelial stem cells [86]. The limbal epithelial stem cells [118, 119] are responsible for the regeneration of the anterior corneal epithelium and help maintain its transparency. These cells are exclusively found in Vogt’s palisades, which provide a specialized microenvironment for their renewal and proliferation [86]. Dua [88] and Dua et al. [89] reported that a deficiency of these cells in humans can lead to corneal opacification due to conjunctivalization and vascularization of the transparent cornea. According to Sanchez and Daniels [86], there are specific disease entities in domestic animals, such as Canine Herpes Virus-1 suggesting a diagnosis of neurotrophic keratitis [90], that can lead to vascularization, conjunctivalization, and/or pigmentation of the corneal surface. In humans, a deficit in limbal epithelial stem cells can result from genetic diseases, chronic inflammation, chemical exposure, heat, or radiation. However, in domestic and wild mammals, the incidence of conjunctivalization in corneal diseases is challenging to define due to the limited use of methods like impression cytology in veterinary ophthalmology [61, 86].

In terrestrial carnivores, the shape of the pupil varies depending on the species’ habitat, activity pattern (diurnal or nocturnal), and its adaptation to multifocal optical systems, as well as whether they are considered “predators” or “prey” (with daytime predators and nocturnal predators, diurnal-nocturnal predators, and crepuscular vertebrates aiming to maximize light-gathering ability) [61,91,92,129,135,136]. Banks et al. [91], cited by Carvalho et al. [70], reported that horizontal pupils are exclusively found in prey species, regardless of their activity pattern. In our analyzed red panda (a nocturnal and crepuscular animal), the pupil was horizontally ovoid, similar to that of the ring-tailed coati (mainly diurnal) and ferrets [70, 93]. Kitchener et al. [121] noted that in the subfamily Mustelinae, the pupil tends to be close to horizontal slits, likely corresponding to the horizontal visual streak characterized by high densities of photoreceptors in their retina and adjacent ganglion cells. In contrast, the pupil of the common raccoon, a nocturnal animal, is wide [94].

Macroscopically, the *tapetum lucidum* in the examined red panda appears milky-colored and has a crescent shape. In contrast, in the kinkajou (*Cercoleptes caudivolvulus*), it is described as yellowish-green, while in the ring-tailed coati, it is bright green with yellowish patches [79]. Carvalho et al. [70] noted that the *tapetum lucidum* in juvenile ring-tailed coatis is greenish-blue, whereas in adults it is yellow. This variation in color indicates not only age-related differences but also intra-species color variations [137]. Ollivier et al. [95] reported that the color of the *tapetum lucidum* can vary based on species, breed, age, coat color, and the amount of pigmentation in the eye and skin.

In the examined red panda, the lens was a biconvex round body, similar to that of the domestic dog, South African painted dog, and Asiatic black bear [44, 60, 61]. In contrast, in the crab-eating fox, the lens was elliptical, smaller, and more rounded than in the aforementioned animals [62]. Malmström and Kröger [92] suggest that in terrestrial mammals, the type of lens and its relationship to the pupil are important factors for adaptation to multifocal optical systems, particularly for nocturnal and crepuscular animals, to achieve maximum light-gathering ability. In the examined red panda, the optic disc was oval-shaped, whereas in the families Mustelidae and Procyonidae, the optic disc is described as round. The fundus exhibits varying colors depending on the species of the animal, even within a given family [79].

Histologically, the red panda possesses a choroidal *tapetum lucidum cellulosum*, as do members of the family Mustelidae [95–97]. A characteristic feature of the choroidal *tapetum lucidum cellulosum* is the variable number of cell layers forming the *tapetum lucidum*, depending on the species. For instance, in the examined red panda, there were 5 to 9 layers of cells, while in the domestic ferret, there were 5 to 7 cell layers, and in the ranch mink, only 2–3 layers were present [95]. In the red panda, this *tapetum* was composed of loosely packed oval cells of varying diameters with large round nuclei. The *tapetum* was surrounded by fine collagen fibers and numerous clusters of melanin granules. The structure of the *tapetum lucidum* in red pandas differs from that in the Canidae or Felidae families, where the *tapetum lucidum* cells have transversely arranged capillary vessels. Such vessels were not found in our red pandas. We can hypothesize that the histological structure of the *tapetum lucidum* in the red panda is characteristic of the family Ailuridae. However, to fully confirm this, further studies should be conducted on a larger number of individuals. On the other hand, the histological structure of the choroidal *tapetum lucidum cellulosum* in the ranch mink is intriguing due to features of tapetal cell degeneration, such as a non-regular internal arrangement of membranes [95, 96, 121]. Ollivier et al. [95] reported that the cause of this degeneration is unknown but could be related to dietary insufficiencies in specific animals or perhaps to the genetics of inbreeding.

## Conclusion

A detailed characterization of the orbital region, eyelids, orbital glands, and eye tunics in the red panda, the sole living representative of the Ailuridae family, has not been undertaken until now. Characteristics observed in the red panda include the presence of: a single ethmoid foramen, a fossa for the lacrimal sac, a trochlear fovea, a caudal lacrimal process, an external lacrimal fossa, a weak orbitotemporal crest. Additionally, there are simple alveolar glands in both upper and lower eyelids, a palpebral part of the lacrimal gland in the upper eyelid, and a superficial gland of the third eyelid. The lacrimal gland consists of a branched acinar complex producing mucous secretion. Furthermore, palisades of Vogt are present in the corneal epithelial limbus. Macroscopically, a milky-colored crescent-shaped *tapetum lucidum*, and histologically, a choroidal *tapetum lucidum* cellulosum composed of 5 to 9 layers of loosely packed oval cells, have been observed. Additionally, features common to both the Ailuridae family and the clade Musteloidea and terrestrial Caniformia have also been noted.

## Data Availability

The authors confirm that our article type does not require a data availability statement.

## Abbreviations

PAS: Periodic Acid–Schiff
AB pH 1.0: Alcian blue pH 1.0
AB pH 2.5: Alcian blue pH 2.5
HDI: Hale’s Dialyzed Iron Stain
UE: Upper Eyelid
LE: Lower Eyelid
SGTE: Superficial Gland Of The Third Eyelid
TE: Third Eyelid
LG: Lacrimal Gland
